# Reassessing antimicrobial resistance governance in Latin America: the case for antimicrobial consumption as the primary metric of implementation success

**DOI:** 10.3389/fpubh.2026.1781566

**Published:** 2026-04-15

**Authors:** Esteban Zavaleta-Monestel, Jose Pablo Diaz-Madriz, Jeaustin Mora Jiménez, Sebastián Arguedas-Chacón

**Affiliations:** 1 Research Department, Hospital Clínica Bíblica, San José, Costa Rica; 2 Pharmacy Department, Hospital Clínica Bíblica, San José, Costa Rica

**Keywords:** antimicrobial consumption, antimicrobial resistance, antimicrobial stewardship, AWaRe classification, electronic prescribing, Latin America

## Introduction: misaligned incentives in AMR governance

Antimicrobial resistance (AMR) is widely recognized as one of the most serious global public health threats, with particularly acute implications for Latin America given high infectious disease burdens, fragmented health systems, and persistent inequities in access to care (1). Globally, bacterial AMR was associated with an estimated 4.95 million deaths in 2019, including 1.27 million deaths directly attributable to resistance, placing AMR among the leading causes of infectious mortality worldwide. These patterns also point to the role of health system governance in shaping antimicrobial use across sectors. Thus, antimicrobial resistance should not be understood solely as an epidemiological problem, but also as a matter of governance (2).

Within the WHO Region of the Americas, the burden is similarly substantial. In 2019, approximately 569,000 deaths were associated with bacterial AMR, including around 141,000 deaths directly attributable to resistance, pointing to the scale of the problem relative to regional health-system capacity (3). In response, countries across Latin America have invested considerable political and institutional effort in aligning with the World Health Organization's Global Action Plan on AMR through the adoption of national action plans and formal governance structures. However, the adoption of national action plans does not necessarily translate into effective implementation or measurable impact on antimicrobial use (4).

Although this alignment appears to indicate progress, actual reductions in the burden of resistant infections have been limited and inconsistent. Comparative analyses of national AMR strategies reveal widespread policy adoption alongside persistent gaps in implementation and outcomes, especially in middle-income countries, reflecting broader implementation challenges documented across low- and middle-income settings (5, 6). This disconnect indicates that the core issue is not a lack of awareness or commitment, but rather a fundamental flaw in the criteria used to define and assess AMR governance success (6).

Current approaches to AMR governance systematically overemphasize the existence of plans, committees, and regulatory documents, while underestimating the capacity required to influence antimicrobial use in practice. This misalignment creates an illusion of implementation: formal compliance gives the appearance of progress, even as the behaviors driving resistance persist. Antimicrobial consumption, rather than formal policy adoption, should therefore serve as the primary metric for evaluating AMR governance success in Latin America. In this context, antimicrobial consumption should be understood at the population level, capturing use across both public and private healthcare sectors, and measured using standardized indicators such as defined daily doses (DDD) per 1,000 inhabitants per day, complemented by classifications such as AWaRe to assess the appropriateness of use. This article argues that antimicrobial consumption should be the primary operational metric against which AMR governance success is evaluated in Latin America, and that all subsequent policy, regulatory, and stewardship discussions should be interpreted in relation to this central premise. Within this framework, political commitment should not be considered the primary limiting factor.

This suggests that the challenge is not primarily driven by a lack of political commitment, as countries across Latin America have demonstrated sustained engagement with antimicrobial resistance policy frameworks and governance structures (7). Nevertheless, regional analyses reveal persistent implementation constraints, including limited surveillance capacity, weak regulatory enforcement, and fragmented health systems, which continue to undermine the effective control of antimicrobial use (8).

National action plans are appealing governance instruments due to their visibility, auditability, and international recognition . In contrast, interventions that directly influence antimicrobial consumption, such as enforcing prescription regulations, regulating private-sector prescribing, or restricting access to broad-spectrum agents, are politically sensitive, administratively complex, and often unpopular. This pattern is evident across multiple countries in Latin America, including Brazil, Colombia, and Argentina, where national action plans have been formally adopted, yet persistent challenges in surveillance capacity, regulatory enforcement, and intersectoral coordination continue to limit their effective implementation (7). When governance success is measured primarily by the existence of plans rather than by behavioral change, incentives tend to favor symbolic compliance over substantive operational control (8).

**Figure 1:**
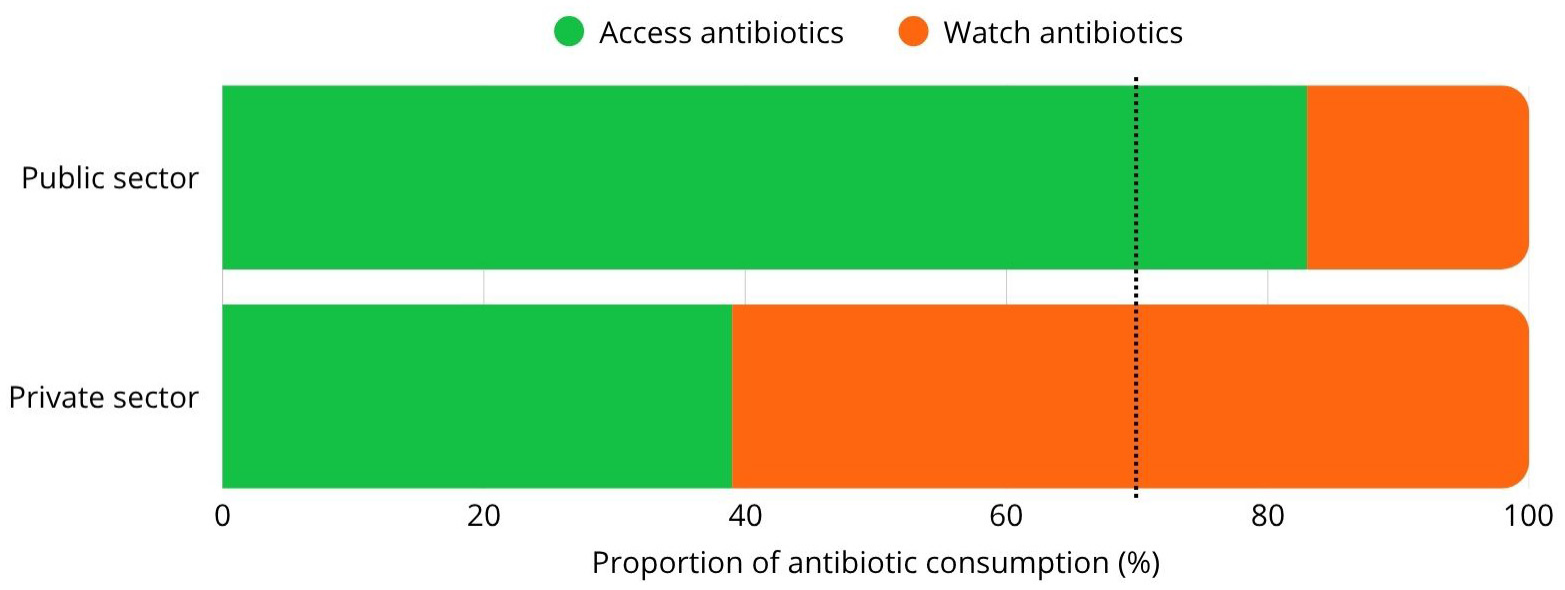
Conceptual framework for reorienting AMR governance in Latin America toward antimicrobial consumption as the primary metric of implementation success.

Recent country-specific evidence reinforces this pattern. In Brazil, antimicrobial consumption trends and stewardship capacity continue to reveal structural implementation gaps despite formal policy adoption (9). In Argentina, country-level antimicrobial consumption measurement has informed concrete regulatory action, illustrating that surveillance can function not only as a descriptive tool but also as a trigger for governance reform (10). Multi-country data from 13 nations in Latin America and the Caribbean further show wide heterogeneity in national antimicrobial consumption, underscoring the absence of shared regional accountability standards and the limited comparability of current governance performance (10). In parallel, a recent global evaluation of AMR governance across 193 countries found that implementation and monitoring indicators consistently lag behind policy design, particularly outside the human health sector. As shown in Figure 1, this disconnect reinforces that plan adoption alone cannot substitute for performance-based governance (11).

This dynamic reflects a failure of incentive design rather than a lack of intent. These limitations do not diminish the significant efforts undertaken by countries in the region; instead, they highlight that current governance models are inadequate for consistently translating commitment into effective control of antimicrobial use.

## Antimicrobial consumption is systematically sidelined

Antimicrobial consumption lies at the heart of resistance dynamics. Global studies show substantial increases and convergence in antibiotic use over time, reinforcing the central role of consumption patterns in the emergence and spread of resistance (12). Evidence consistently demonstrates a positive association between antimicrobial consumption and resistance rates, with higher levels of antibiotic use contributing to the emergence and spread of resistant pathogens across both hospital and community settings (12). This relationship has gained increasing policy relevance through standardized antimicrobial consumption surveillance and AWaRe-informed monitoring frameworks, which allow countries to move from descriptive reporting toward measurable accountability for prescribing patterns. Yet despite this well-established relationship, antimicrobial consumption remains weakly integrated into many AMR governance assessments.

In Latin America, persistent gaps in the measurement and comparability of antimicrobial consumption limit accountability and policy learning (13). WHO's Global Antimicrobial Resistance and Use Surveillance System (GLASS) was expanded to include antimicrobial consumption precisely to address this gap, yet comprehensive national reporting—particularly across both public and private sectors—remains the exception rather than the norm (14). Although 126 countries participate in GLASS, reporting of antimicrobial consumption remains uneven and incomplete, reflecting important gaps in surveillance capacity and data representativeness (15).

These challenges are frequently characterized as technical, such as limitations in information systems or analytical capacity. While these constraints are genuine, they are also inherently political. Measuring consumption reveals prescribing patterns, dispensing practices, regulatory oversight, and beneficiaries. Consumption data illuminate private-sector dynamics, enforcement shortcomings, and market incentives that many administrative frameworks tend to overlook.

## What changes when consumption is measured: the Costa Rica case

Costa Rica is presented as an illustrative case due to the availability of comprehensive antimicrobial consumption data integrating both public and private healthcare sectors, which remains relatively uncommon in Latin America (16). In Costa Rica, comprehensive measurement of antimicrobial consumption made visible governance gaps that are not captured by metrics focused solely on the existence of plans or formal regulatory frameworks. A national retrospective analysis for 2019 estimated total antimicrobial consumption at 14.32 defined daily doses per 1,000 inhabitants per day (DDD/1,000 inhabitants/day), integrating for the first time data from both the public and private sectors to generate a national estimate of antimicrobial use (16).

Public-sector consumption was estimated using dispensation data from the Costa Rican Social Security System (CCSS), which records antimicrobials dispensed within publicly funded healthcare facilities, while private-sector consumption was reconstructed from aggregated pharmaceutical sales data provided by IQVIA, covering private community pharmacies and hospital pharmacies belonging to private healthcare institutions and explicitly excluding any governmental dispensing. These sources reflect two distinct and non-overlapping antimicrobial supply and distribution circuits within the Costa Rican health system, allowing their integration to estimate national antimicrobial consumption without risk of overlap or double counting (16).

**Figure 2:**
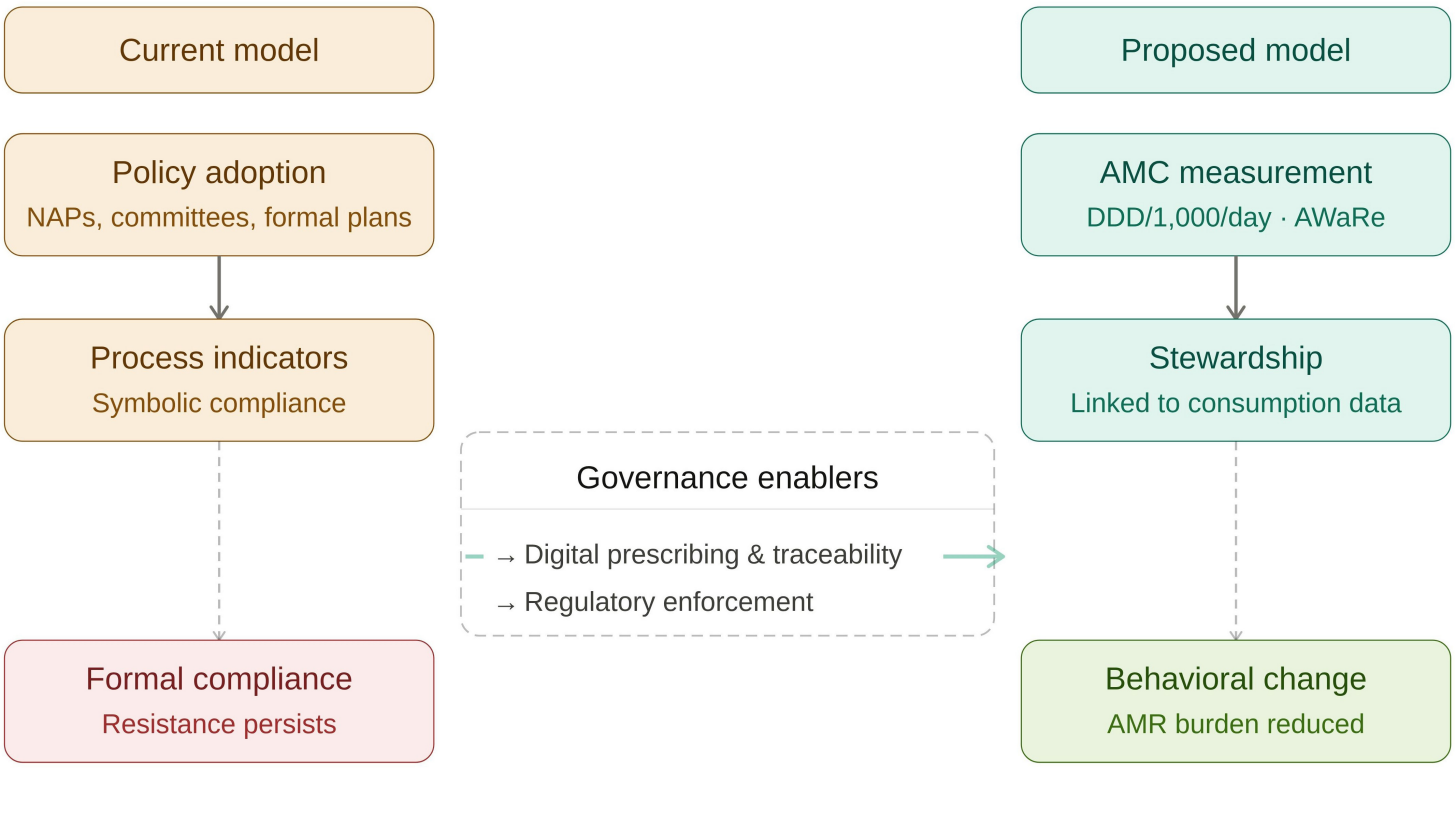
Distribution of AWaRe antibiotic categories by healthcare sector in Costa Rica, 2019. AMC, antimicrobial consumption; DDD, defined daily doses; AWaRe, WHO antibiotic classification.

Although the public sector accounted for the majority of total consumption, sector-specific analysis revealed markedly different use patterns. In the public sector, 83.0% of consumption corresponded to antibiotics classified in the WHO AWaRe *Access* category, reflecting institutional prescribing controls and restrictive mechanisms linked to level of care and medical specialty. In contrast, in the private sector, 61.0% of consumption consisted of *Watch* antibiotics, characterized by broader spectra and higher resistance-selection potential, with greater reliance on macrolides, fluoroquinolones, and broad-spectrum antibiotic combinations (16). Figure 2 illustrates the distribution of AWaRe antibiotic categories across the public and private sectors in Costa Rica.

The measurement process also exposed structural challenges with direct implications for AMR governance, including fragmentation of information systems, limited regulatory visibility of private-sector use, and the inability to consistently differentiate outpatient from inpatient consumption. These limitations are not merely technical, but reflect institutional and regulatory constraints that condition the state's capacity to exert effective and uniform control over antimicrobial use across sectors. In this context, the Costa Rica case illustrates how systematically incorporating antimicrobial consumption as an evaluative metric shifts the governance debate from policy alignment to accountability, enforcement, and the design of incentives that directly shape prescribing behavior (16).

While the Costa Rican case provides a valuable illustration of how comprehensive antimicrobial consumption data can reframe governance debates, its findings should be interpreted with caution when considering regional applicability. Costa Rica's capacity to integrate public and private sector data reflects a specific configuration of institutional infrastructure—including a unified social security dispensing system and access to commercial pharmaceutical sales databases—that is not uniformly available across Latin America (16). Countries at earlier stages of data maturity need not replicate this model as a precondition for initiating consumption-based governance. A phased approach is operationally feasible: beginning with public-sector dispensing data available within national formulary or social security systems, and progressively incorporating private-sector pharmaceutical sales information through partnerships with commercial data providers. Evidence from a multi-country analysis across 13 nations in the Americas confirms that even partial national estimates are useful for identifying prescribing patterns, comparing temporal trends, and benchmarking performance against peers (10). PAHO's antimicrobial consumption surveillance methodology, developed in collaboration with CUFAR (University Center for Pharmacology, National University of La Plata, Argentina), is designed to accommodate countries at different levels of data infrastructure through standardized training and technical guidance, making incremental adoption feasible across the region (10).

## The resistance patterns are already undermining healthcare systems

The consequences of weak implementation are no longer abstract. Across Latin America, healthcare systems are increasingly shaped by a relatively small set of highly consequential resistant pathogens that drive morbidity, mortality, and costs.

Regional surveillance consistently identifies carbapenem-resistant Gram-negative organisms, including *Klebsiella pneumoniae* and *Acinetobacter baumannii*, among the most frequently reported causes of healthcare-associated infections in hospital settings (17). These pathogens are associated with prolonged hospitalization, excess mortality, and reliance on last-line or toxic therapies, placing sustained pressure on already constrained systems (18).

Globally, Gram-negative bacteria account for nearly 70% of deaths directly attributable to bacterial AMR, underscoring their disproportionate contribution to resistance-related mortality and health system burden (2). Resistant bloodstream infections, which are routinely reported through global surveillance systems, remain a major driver of excess mortality and healthcare costs (14).

These resistance patterns are unevenly distributed, disproportionately impacting under-resourced facilities, peripheral hospitals, and fragmented private-sector settings—contexts that often fall outside the scope of national governance assessments. Their persistence reflects not microbiological inevitability, but upstream governance failures, including unchecked consumption, delayed detection, and weak regulatory signals.

## Structural and regulatory constraints shaping antimicrobial use

Several structural features of health systems in Latin America magnify these resistance dynamics. Weak laboratory infrastructure and delayed diagnostics sustain empiric prescribing and prolonged use of broad-spectrum agents, undermining stewardship at the point of care (19).

The political economy of antimicrobial governance in private health markets presents a distinct and underanalyzed set of constraints. In Latin American mixed health systems, pharmaceutical supply chains, private pharmacy retail networks, and private prescribing operate under commercial incentives that are structurally misaligned with antimicrobial consumption reduction goals. Self-medication rates of 14–26% have been documented across the region, driven by widespread over-the-counter antibiotic access that persists despite formal prescription-only regulations in most national legal frameworks (8). Private-sector actors—including pharmaceutical wholesalers, pharmacy chains, and private hospital systems—face no systematic obligation to report dispensing data to health ministries in the majority of Latin American countries, generating regulatory blind spots that reflect institutional architecture as much as technical limitation. Pharmaceutical manufacturers and distributors retain commercial interests in maintaining market share for broad-spectrum Watch-category agents, creating incentives that may run counter to stewardship goals. Governing antimicrobial consumption across mixed markets therefore requires not only information system improvements, but explicit regulatory negotiations over what private-sector actors must report, to whom, and with what consequences for non-compliance—dimensions that current national action plans rarely address with operational specificity.

These constraints are frequently acknowledged in national strategies yet are seldom addressed through concrete operational measures. Governance frameworks often describe these challenges without resolving them, continuing a cycle in which persistent problems remain unaddressed and accountability for change is lacking.

A comprehensive reassessment of AMR governance around consumption must also acknowledge its One Health dimensions. The Global Action Plan on AMR and its quadripartite framework—encompassing WHO, FAO, WOAH, and UNEP—formally recognize that antimicrobial use in food animals, aquaculture, and crop production contributes to resistance selection pressure with cross-sectoral human health consequences. In practice, however, published AMR interventions in Latin America and the Caribbean have concentrated almost exclusively on human health settings: a recent scoping review identified that 75 of 82 published studies addressed human health while only 7 addressed animal health sectors (8). A parallel assessment of One Health governance across countries in Latin America and the Caribbean found that coordination on antimicrobial management and disposal ranked among the lowest-scoring domains, indicating that intersectoral regulation of non-human antimicrobial use remains underdeveloped in the region (20). This paper focuses on human health antimicrobial consumption as the primary governance metric—an analytically necessary scope boundary—but future governance frameworks should progressively incorporate veterinary and agricultural consumption data through tools such as WOAH's ANIMUSE platform and intersectoral regional surveillance mechanisms, so that consumption-based accountability does not remain confined to a single sector.

## Digital health and electronic prescribing: from visibility to enforceability

Digital health interventions, particularly electronic prescribing and dispensing systems, are increasingly promoted as solutions to antimicrobial misuse. In Costa Rica, national planning explicitly links electronic prescribing to improved traceability, monitoring, and enforcement for systemic antimicrobials (21). Argentina has also introduced regulatory and policy measures intended to strengthen oversight of antimicrobial prescribing (22).

The value of electronic prescribing does not reside in digitalization alone, but in the capabilities that digital systems enable. When effectively designed and governed, electronic prescription platforms can provide real-time visibility of antimicrobial use, facilitate integration between prescribing, diagnostics, and dispensing, and support feedback mechanisms essential for stewardship and regulation. These systems can also reduce opportunities for informal dispensing and prescription reuse, which are continuing obstacles in several Latin American contexts.

Beyond traceability, the stewardship literature also shows that digital systems can improve prescribing appropriateness, strengthen linkage between prescription and dispensing records, and support the audit-and-feedback loops required for antimicrobial stewardship. A systematic review of antimicrobial stewardship programs across Latin America and the Caribbean found that digital decision-support tools and monitoring systems are increasingly being adopted at the institutional level, but their integration into national regulatory and accountability frameworks remains weak; hospital leadership support and monitoring were among the least-developed stewardship components in the region (23). Regional experiences further suggest that electronic prescribing becomes meaningful as governance infrastructure only when paired with institutional capacity, standardized consumption measurement, and enforceable accountability mechanisms. In this sense, digitalization should be understood not as an endpoint, but as an enabling infrastructure for consumption-based governance (24).

Digital systems are not politically neutral; they alter power dynamics by making prescribing practices transparent to regulators, payers, and health authorities. Without clear governance arrangements specifying data access, oversight, and consequences, electronic prescribing may become a surveillance tool lacking enforcement authority. Digital health should thus be conceptualized as accountability infrastructure rather than merely a technological solution.

## Stewardship without measurement is performative

Antimicrobial stewardship is widely promoted as a cornerstone of AMR control, but its effectiveness depends on the governance environment in which it operates. Stewardship challenges the autonomy of prescribing, professional norms, and market incentives; it is therefore inherently political, not merely technical (25).

Without routine measurement of consumption, digital traceability, and regulatory support, stewardship risks becoming performative: endorsed in principle and highlighted in reports, yet marginal in daily clinical practice. Governance centered on consumption is not merely an enhancement to stewardship; it is the prerequisite for stewardship to function beyond aspiration (26).

## Conclusion

Latin America has developed multiple AMR strategies, but still lacks governance frameworks that consistently prioritize the control of antimicrobial use over the appearance of preparedness. As long as antimicrobial consumption remains peripheral to the definition of success, resistance will continue to outpace policy interventions.

Re-centering antimicrobial consumption as the primary metric of AMR governance requires the routine and standardized measurement of use across both public and private sectors, the integration of consumption indicators into national accountability frameworks, and the alignment of stewardship efforts with measurable outcomes rather than process indicators alone. PAHO, WHO GLASS surveillance frameworks, national regulatory authorities, and regional stewardship networks can support this transition by harmonizing standards, strengthening technical capacity, and promoting clearer accountability mechanisms.

Carbapenem-resistant Gram-negative pathogens, ESBL-producing Enterobacterales, and persistent MRSA are no longer emerging threats, but entrenched realities shaping mortality, costs, and inequities across healthcare systems in the region. Re-centering antimicrobial consumption within AMR governance is therefore not a radical shift, but a necessary correction. Until antimicrobial use is governed with the same rigor as policy alignment, AMR strategies in Latin America will continue to appear successful on paper while failing in practice.
